# A Variant Mimicking Hyperphosphorylated 4E-BP Inhibits Protein Synthesis in a Sea Urchin Cell-Free, Cap-Dependent Translation System

**DOI:** 10.1371/journal.pone.0005070

**Published:** 2009-03-31

**Authors:** Nathalie Oulhen, Sandrine Boulben, Michael Bidinosti, Julia Morales, Patrick Cormier, Bertrand Cosson

**Affiliations:** 1 UPMC Univ Paris 06, UMR 7150, Equipe Traduction Cycle Cellulaire et Développement, Station Biologique de Roscoff, Roscoff, France; 2 CNRS, UMR 7150, Station Biologique de Roscoff, Roscoff, France; 3 Université Européenne de Bretagne, Bretagne, France; 4 Department of Biochemistry and Rosalind and Morris Goodman Cancer Centre, McGill University, Montreal, Quebec, Canada; National Institute on Aging, United States of America

## Abstract

**Background:**

4E-BP is a translational inhibitor that binds to eIF4E to repress cap-dependent translation initiation. This critical protein:protein interaction is regulated by the phosphorylation of 4E-BP. Hypophosphorylated 4E-BP binds to eIF4E and inhibits cap-dependent translation, whereas hyperphosphorylated forms do not. While three 4E-BP proteins exist in mammals, only one gene encoding for 4E-BP is present in the sea urchin genome. The protein product has a highly conserved core domain containing the eIF4E-binding domain motif (YxxxxLΦ) and four of the regulatory phosphorylation sites.

**Methodology/Principal Findings:**

Using a sea urchin cell-free cap-dependent translation system prepared from fertilized eggs, we provide the first direct evidence that the sea urchin 4E-BP inhibits cap-dependent translation. We show here that a sea urchin 4E-BP variant, mimicking phosphorylation on four core residues required to abrogate binding to eIF4E, surprisingly maintains physical association to eIF4E and inhibits protein synthesis.

**Conclusions/Significance:**

Here, we examine the involvement of the evolutionarily conserved core domain and phosphorylation sites of sea urchin 4E-BP in the regulation of eIF4E-binding. These studies primarily demonstrate the conserved activity of the 4E-BP translational repressor and the importance of the eIF4E-binding domain in sea urchin. We also show that a variant mimicking hyperphosphorylation of the four regulatory phosphorylation sites common to sea urchin and human 4E-BP is not sufficient for release from eIF4E and translation promotion. Therefore, our results suggest that there are additional mechanisms to that of phosphorylation at the four critical sites of 4E-BP that are required to disrupt binding to eIF4E.

## Introduction

Modulation of the protein synthesis machinery is important in the regulation of gene expression in eukaryotes [1]. Although sea urchin eggs contain all of the necessary components for translational activity [2], protein synthesis is low in unfertilized eggs and is stimulated rapidly following fertilization [3], [4]. This change results from an increase of the translation initiation rate of masked maternal mRNAs [5], [6], independently of mRNA transcription and ribosome biogenesis [3], [7]. Cap-dependent translation is highly regulated following fertilization and is involved in the onset of the first mitotic division of sea urchin embryos [8]. Translation initiation in eukaryotes requires a highly conserved battery of protein complexes [9]. Consistently, we recently identified in the sea urchin genome [10] an ortholog of each mammalian translation initiation factor [11].

Among the protein synthesis machinery components, the eukaryotic Initiation Factor 4E (eIF4E) is a major target for the control of cap-dependent translation initiation [12]. eIF4E is a cap-binding protein that interacts with the mRNA 5′-cap structure, m^7^GpppN (where N is any nucleotide). eIF4E functions by recruiting eukaryotic Initiation Factor 4G (eIF4G), a large scaffolding protein that acts as docking site for several proteins including eukaryotic Initiation Factors 4A (eIF4A) and 3 (eIF3). Consequently, eIF4E bridges the ribosome and the mRNA [13]. eIF4G binds also to the poly(A)-binding protein (PABP), which interacts with the mRNA poly(A) tail, and this promotes a closed loop conformation of the mRNA, which is thought to stabilize the interaction of the cap-binding complex and to stimulate translation of polyadenylated RNAs [14]. eIF4E is the limiting factor in translation initiation under most circumstances and plays an important role in the regulation of the cell cycle [15]–[17], and is also thought to have a role during reproduction and embryogenesis [18].

The 4E-binding proteins [4E-BP; also called phosphorylated heat-and-stable protein, insulin-stimulated (PHAS-I)] compete with eIF4G for a mutually exclusive binding site on the dorsal surface of eIF4E by way of a shared eIF4E-recognition motif, YxxxxLФ (where x is any amino acid and Ф is an aliphatic residue, usually L, M or F) [19] and consequently inhibit cap-dependent translation [20]. Binding of 4E-BPs to eIF4E is dependent upon the phosphorylation status of 4E-BPs [21], [22]. Hypophosphorylated 4E-BPs bind to eIF4E and inhibit cap-dependent translation, whereas hyperphosphorylated forms do not [13]. However, the relationship between the multiple states of 4E-BPs phosphorylation and their dissociation from eIF4E is not fully understood [23]. Three 4E-BP proteins (4E-BP1, 4E-BP2 and 4E-BP3) exist in mammals [21], [24], [25]. The most thoroughly studied of these is 4E-BP1, for which multiple and hierarchical phosphorylation events are required for release from eIF4E [26]. 4E-BP1 phosphorylation has been reported on multiple sites: T37, T46, S65, T70, S83, S101 (all in an S/TP consensus), and S112 (a SQ site numbered according to human 4E-BP1) (review in Raught and Gingras [27]). Phosphorylation of S101 is constitutive and has been proposed to be required for efficient phosphorylation of S65 [28]. The function of phosphorylation at S83 and S112 is not clear [27]. 4E-BP1 phosphorylation on the four other sites proceeds in a hierarchical manner: phosphorylation on both T37 and T46 is required as a priming event for subsequent phosphorylation of T70, which precedes phosphorylation of S65 and subsequent dissociation from eIF4E [26]. A large body of evidence indicates that phosphoinositide 3′-kinase (PI3K) and FKBP12 rapamycin-associated protein/mammalian target of rapamycin (FRAP/mTOR) signaling kinases effect the phosphorylation and release of 4E-BP1 from eIF4E [29]. Recently, it has been proposed that 4E-BP1 phosphorylation may play another role in the regulation of protein synthesis, promoting the conversion of 4E-BP1 to alternative polyubiquitinated forms that may be involved in the increase of the turnover of this translational repressor [30].

The single sea urchin 4E-BP ortholog harbours a highly conserved core domain containing the eIF4E-binding motif and the phosphorylation sites (T37, T46, S65 and T70, numbered according to human 4E-BP1) [11]. Following fertilization of the sea urchin eggs, 4E-BP is rapidly phosphorylated and degraded [8], [31]. The release of eIF4E from its translational repressor 4E-BP and the subsequent formation of the eIF4E/eIF4G complex correlate with the rapid increase of protein synthesis triggered by fertilization and are required for the onset of the first mitotic division of embryonic development [32], [33]. The dissociation of 4E-BP from eIF4E and its degradation are inhibited by rapamycin, suggesting a role for protein phosphorylation in these two processes.

Using a cell-free translation system, the activity of several translation initiation factors has been shown to be enhanced upon fertilization [34]–[37], consistent with the increase of the rate of translation initiation in fertilized eggs. It was suggested that association of eIF4E with eIF4G and eIF4A was repressed by an inhibitor of unknown identity in unfertilized eggs [38]. Previous work from our laboratory demonstrated a correlation between eIF4E release from its repressor 4E-BP and the rise of protein synthesis that occurs shortly following egg fertilization [31]. In the present study, we characterize the translational repression activity of the sea urchin 4E-BP using a cell-free translation system that mimics the increase of the rate of translation initiation in fertilized eggs.

## Results

### Isolation and characterization of the cDNA encoding the *Spharechinus granularis* Sg4E-BP

**Figure 1:**
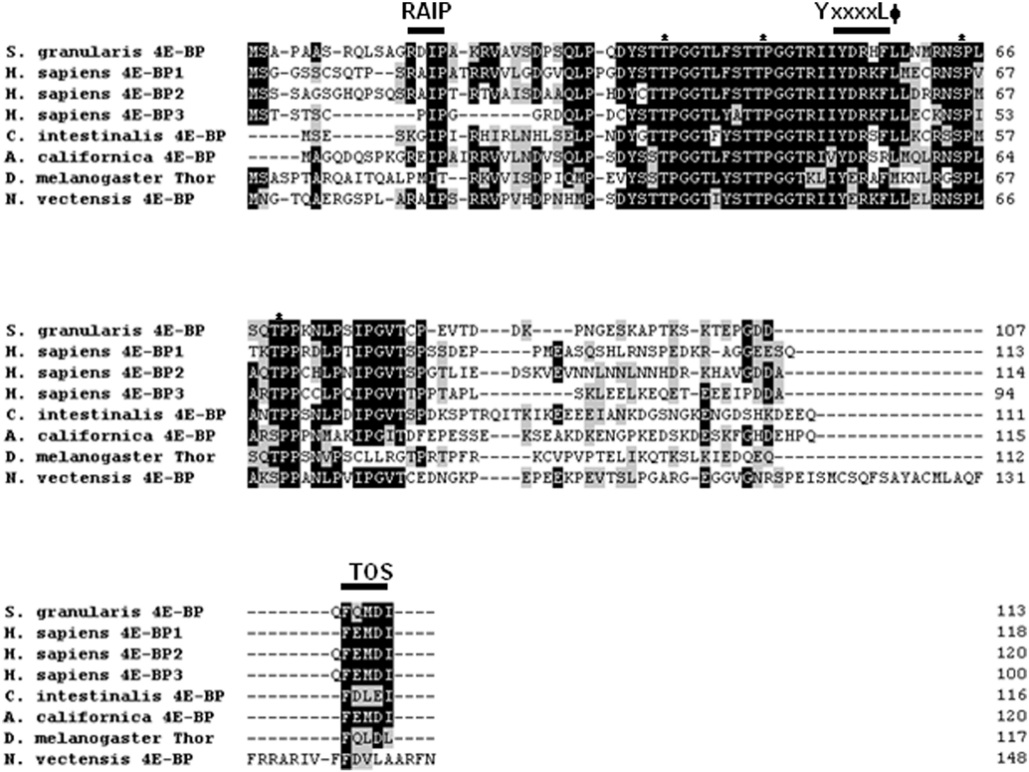
Amino acid sequences of 4E-BP proteins. Sg4E-BP protein was predicted from the cDNA and aligned with the three human 4E-BPs, *Ciona intestinalis* 4E-BP, *Drosophila melanogaster* Thor, *Aplysia californica* 4E-BP and *Nematostella vectensis* 4E-BP. Accession numbers of human proteins are: 4E-BP1 (NP_004086), 4E-BP2 (Q13542), 4E-BP3 (NP_003723) on NCBI. The Accession number is NP_477295 for *Drosophila melanogaster* (NCBI), 299279 for *Ciona intestinalis* (http://genome.jgi-psf.org/Cioin2/) and SB_47700 for *Nematostella vectensis* (http://www.stellabase.org/). The *Aplysia californica* sequence 4E-BP was obtained from [44]. The four residues known to be phosphorylated on human 4E-BPs and conserved on sea urchin are indicated by stars. Identical and conserved amino acid residues are on black and grey background, respectively. The common eIF4E motif indicated by YxxxxLΦ (where x is any amino acid and Φ is an hydrophobic residue), the RAIP motif and the TOS (TOR signalling) site are denoted with a line above.

**Figure 2:**
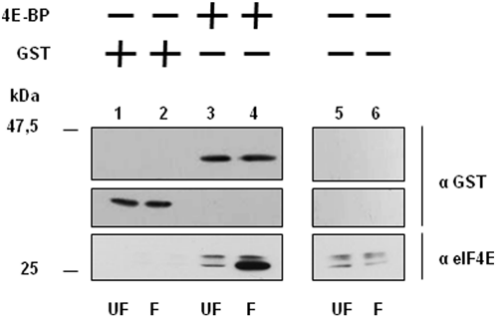
Recombinant GST-Sg4E-BP (4E-BP) interacts with endogenous SgIF4E isoforms in sea urchin extracts. After incubation of the GST alone (lanes 1–2) or the GST-Sg4E-BP protein (lanes 3–4) in extract prepared from unfertilized eggs (UF, lanes 1 and 3) or from 60 minutes post-fertilization embryos (F, lanes 2 and 4), proteins were affinity purified using Gluthatione Sepharose 4B beads, resolved by 15% SDS-PAGE, analysed by immunoblotting and detected by chemiluminescence using an anti-GST antibody (top and intermediate panels) or anti-eIF4E antibody (bottom panels) as described in Materials and Methods. SgIF4E that co-purified with GST-Sg4E-BP (lanes 3 and 4) was compared with the endogenous SgIF4E detected in 10 µg of total protein extracts (corresponding to 0,5% of the volume used for the purification) loaded separately (lanes 5–6).

In order to study the translational inhibitory function of the sea urchin 4E-BP, we isolated and sequenced the cDNA encoding this small protein in the sea urchin *Sphaerechinus granularis*. A polypeptide of 113 amino acids (Fig 1) with a predicted molecular mass of 12,1 kDa was obtained. The protein has a highly conserved core domain containing the eIF4E-binding motif (YxxxxLФ). While the sites homologous to the poorly characterized phosphorylation sites of 4E-BP1 (S83, S101 and S112; numbered according to human 4E-BP1) are strikingly absent, the principal phosphorylation sites (T37, T46, S65 and T70; numbered according to human 4E-BP1) involved in the regulation of eIF4E-binding are conserved in the sea urchin 4E-BP sequence. Two motifs important for protein-protein interactions and that regulate 4E-BP1 phosphorylation, the N-terminal RAIP motif (named according to its amino acid sequence) [39] and the C-terminal TOS site (TOR Signaling) [40], are also present. Full-length *Sphaerechinus granularis* 4E-BP (Sg4E-BP) was cloned and GST-tagged Sg4E-BP fusion protein containing the complete 4E-BP protein sequence was expressed in *E. coli* and purified (see Materials and Methods). We first tested the ability of the GST-Sg4E-BP to associate to sea urchin eIF4E (Fig 2). When incubated in sea urchin extract prepared from unfertilized eggs, the GST-Sg4E-BP fusion protein associated weakly with two endogenous eIF4E isoforms (lane 3) that were also detected in total extract (lanes 5 and 6). Interestingly, when incubated in extract prepared from eggs 60 minutes post-fertilization, the amount of endogenous eIF4E bound to the GST-Sg4E-BP increased dramatically (compare lane 4 with lane 3). This is consistent with an increase in the free eIF4E available to bind to the GST-tagged bait Sg4E-BP, owing to its release from endogenous 4E-BP that is degraded following fertilization [8]. It is interesting to note that the eIF4E isoform that exhibits a greater electrophoretic mobility associates preferentially with the GST-Sg4E-BP protein. Three genes encoding for eIF4E isoforms are present in sea urchin [11]. Whether the two bands retained in the extract correspond to different isoforms or to post translational modification is unknown. These data demonstrate that the sea urchin recombinant 4E-BP polypeptide interacts with endogenous sea urchin eIF4E isoforms. In order to further characterize Sg4E-BP, we analyzed its ability to inhibit cap-dependent translation in a cell-free translation system from sea urchin eggs.

### Sg4E-BP inhibits cap-dependent translation in a sea urchin cell-free cap-dependent translation system

**Figure 3:**
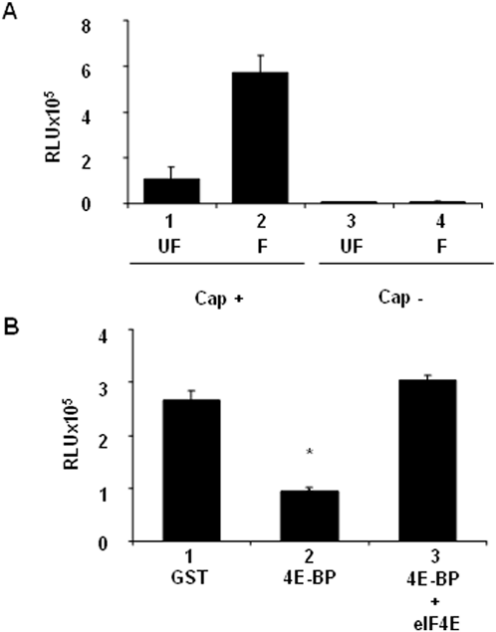
Sg4E-BP inhibits protein synthesis activity in sea urchin cell-free cap-dependent translation system. Cell-free translation system from unfertilized eggs (UF) and 30 minutes post-fertilization embryos (F) were prepared as described in Materials and Methods. (A) Renilla Luciferase activity was measured after addition of capped (Cap+) or uncapped (Cap−) mRNA. Error bars represent the standard deviation (s.d.) of duplicates. (B) Sg4E-BP inhibits cap dependent translation activity and this inhibition is rescued by eIF4E. 100 ng of GST (lane 1), 100 ng of GST-Sg4E-BP alone (4E-BP, lane 2) or preincubated 5 min with 250 ng of GST-mIF4E (4E-BP+eIF4E, lane 3) were added to the fertilized cell free translation system and Luciferase activity was measured as described in Materials and Methods after addition of a capped mRNA encoding Renilla Luciferase. Error bars represent the standard deviation (s.d.) of duplicates. Significance was assessed using Fisher's *F*-test and Student's *t*-test. *P<0.005, significant difference between 4E-BP and GST, and between 4E-BP and 4E-BP+eIF4E.

We first developed a cell-free translation system that was competent for initiating translation of exogenous capped *Luciferase* mRNAs. The system prepared from unfertilized eggs exhibited a modest protein synthesis rate of the exogenous capped mRNA, while that prepared from eggs 30 minutes post-fertilization showed rates of protein synthesis 5 fold higher (Fig 3A, compare lanes 1 and 2). This reflects the increase of protein synthesis rates observed *in vivo* after sea urchin egg fertilization. Importantly, neither cell-free translation system was capable of initiating translation on exogenous uncapped *Luciferase* mRNA (lanes 3–4). Therefore, the cell-free translation system supports cap-dependent protein synthesis activity.

We then found that the GST-Sg4E-BP fusion protein dramatically inhibited protein synthesis activity when measured in a cell-free extract system prepared from fertilized eggs (Fig 3B, compare lanes 1 and 2). Interestingly, translation inhibition by Sg4E-BP was rescued by concomitant addition of a mouse recombinant GST-tagged eIF4E (GST-mIF4E) in the cell-free translation system (lane 3). Taken together, these data provide the first direct demonstration that sea urchin 4E-BP functionally inhibits cap-dependent translation.

### The variant that mimics phosphorylation on T36/T45/S64/T69 of Sg4E-BP associates efficiently with eIF4E and precludes binding of eIF4G to eIF4E *in vitro*

**Figure 4:**
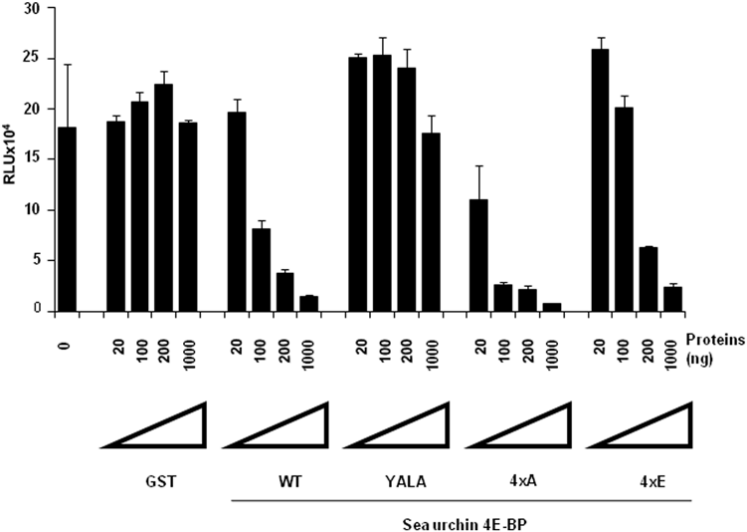
The Sg4E-BP variant mimicking hyperphosphorylation inhibits protein synthesis in sea urchin cell-free cap-dependent translation system. Different amounts (20; 100; 200 or 1,000 ng) of recombinant proteins (GST, GST-Sg4E-BP WT (WT), GST-Sg4E-BP YALA (YALA), GST-Sg4E-BP 4xA (4xA), GST-Sg4E-BP 4xE (4xE)) were added to the fertilized cell-free translation system and Luciferase activity was measured as described in Materials and Methods after addition of the Cap+mRNA encoding Renilla Luciferase. The Luciferase activity is represented in RLU (Relative Light Units). Error bars represent the standard deviation (s.d.) of duplicates.

**Figure 5:**
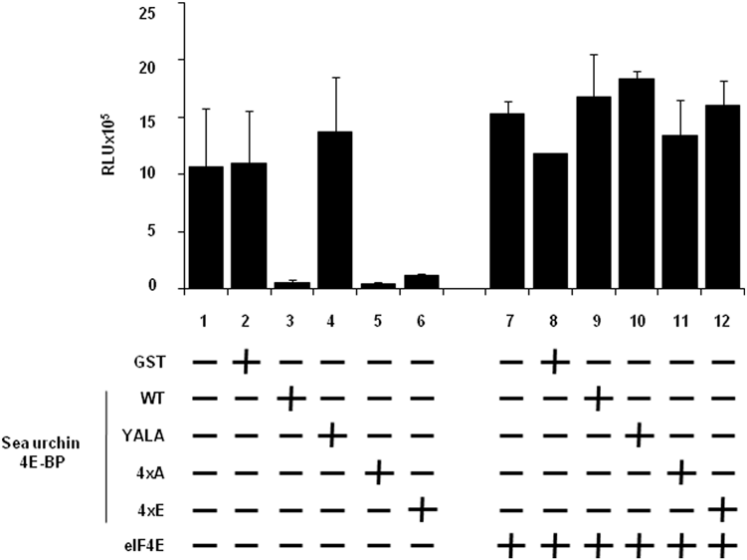
Translation inhibition is rescued by GST-mIF4E. 200 ng of GST recombinant proteins (GST, GST-Sg4E-BP WT (WT), GST-Sg4E-BP YALA (YALA), GST-Sg4E-BP 4xA (4xA), GST-Sg4E-BP 4xE (4xE)) were added to the fertilized cell-free translation system with (lanes 8–12) or without (lanes 2–6) a 5 min pre-incubation with 500 ng of GST-mIF4E (eIF4E) and Luciferase activity was measured as described in Materials and Methods after addition of a cap mRNA encoding Renilla Luciferase. The Luciferase activity is represented in RLU (Relative Light Units). Error bars represent the standard deviation (s.d.) of duplicates.

**Figure 6:**
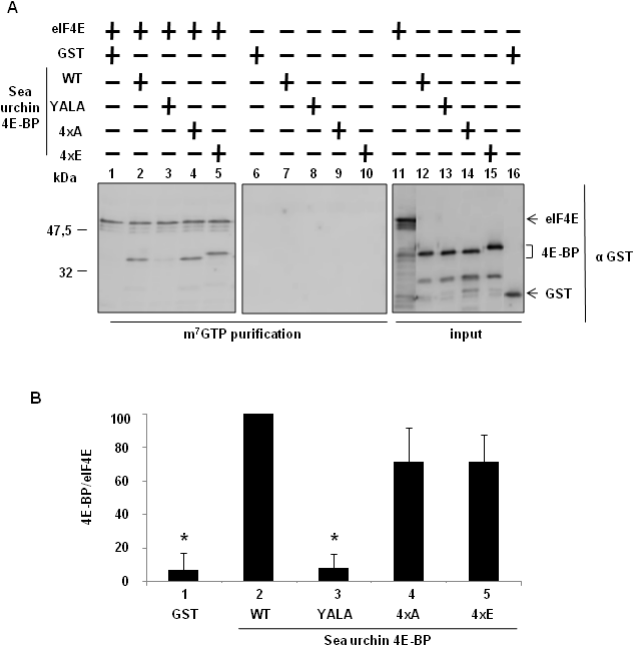
The variant mimicking hyperphosphorylation of Sg4E-BP also binds to eIF4E. (A) The S/T-E Sg4E-BP mutated at all four phosphorylation sites (4xE) binds to eIF4E. GST-mIF4E (eIF4E) was incubated with m^7^GTP sepharose beads (lanes 1–5) and the GST recombinant proteins were added: GST, GST-Sg4E-BP WT (WT), GST-Sg4E-BP YALA (YALA), GST-Sg4E-BP 4xA (4xA), GST-Sg4E-BP 4xE (4xE). Complexes were affinity purified and analysed by Western blot using a GST antibody. Lanes 6–10, GST-mIF4E was omitted as control for binding specificity. Inputs are shown on the right panel (lanes 11–16). They represent 10% of the volume used in the experiment. (B) Binding between the GST recombinant proteins and GST-mIF4E was analysed by quantification of the signals obtained on the Fig 6.A, using Image Quant software. Error bars represent the standard deviation (s.d.) of two experiments. Significance was assessed using Fisher's *F*-test and Student's *t*-test. ^*^ *P*<0.01 significant difference between GST or GST-Sg4E-BP YALA with GST-Sg4E-BP WT.

**Figure 7:**
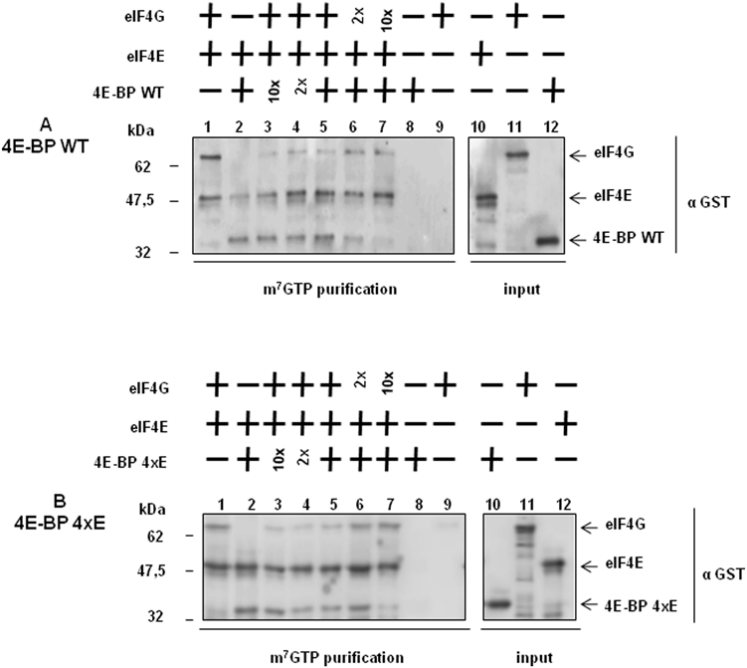
Wild type Sg4E-BP and the S/T-E variant inhibit eIF4E/eIF4G association with the same efficiency. Wild type (A) and the 4xE variant of Sg4E-BP (B) inhibit eIF4E/eIF4G association. The m^7^GTP sepharose beads was incubated (lanes 1–7) or not (lanes 8–9) with GST-mIF4E (eIF4E). Then, recombinant proteins GST-Sg4E-BP WT (WT, A) or GST-Sg4E-BP WT 4xE (4xE, B) and GST-SgIF4G (eIF4G) were added separately for lanes 1–2 and lanes 8–9, and together for other lanes (3-4-5-6-7). We used the same amount of GST-Sg4E-BP and GST-SgIF4G in lane 5, a 2-fold ratio in lanes 4 and 6 and a 10-fold ratio in lanes 3 and 7. Proteins were affinity-purified using m^7^GTP sepharose beads and were analysed by immunoblotting as described in Materials and Methods using an anti-GST antibody. Affinity-purified proteins were compared with the GST-fusion proteins loaded separately (lanes 10–12). Inputs represent 10% of the volume used in the experiment.

The predicted Sg4E-BP protein has a highly-conserved core domain containing four phosphorylation sites and the eIF4E-binding motif (Fig 1). Conservation of these regulatory sites in radial animals such as *N. vectensis* suggests a high level of selection during the evolution of these phosphorylation sites and therefore the maintenance of their functional relevance. We produced a GST-Sg4E-BP variant lacking a competent 4E-binding site, Y53A/L58A (YALA), and also two variants for the four regulatory phosphorylation sites of 4E-BP (corresponding to T37/T46/S65/T70 in human 4E-BP1): the non-phosphorylatable variant T36A/T45A/S64A/T69A (4xA) and the phosphomimetic variant T36E/T45E/S64E/T69E (4xE). We first tested the translation inhibitory activity of the different variants (Fig. 4). Using increasing amounts (20; 100; 200; 1,000 ng) of the different proteins, GST alone, GST-Sg4E-BP (WT: Wild Type), YALA, 4xA and 4xE variants, we tested their capacity to inhibit the translation of a capped *Renilla Luciferase* (R Luc) mRNA in the sea urchin cell-free translation system prepared from fertilized eggs. When added to the active sea urchin cell-free translation system, 200 ng of WT or 4xA variant of Sg4E-BP dramatically impaired the translation of the capped *Luciferase* mRNA whereas the same amount of YALA or GST alone did not affect the translation of the exogenous mRNA. Strikingly, the 4xE variant of Sg4E-BP reduced R Luc expression, in the same proportion as that of the WT and the 4xA variant. The inhibitory effect of the WT and the two variants, 4xA and 4xE, of Sg-4E-BP on cap-dependent translation was completely rescued by addition of the GST-mIF4E fusion protein (Fig 5). Taken together, these data demonstrate that the variant mimicking phosphorylation on T36/T45/S64/T69 of Sg4E-BP is still functional to inhibit protein synthesis in our sea urchin cell-free cap-dependent translation system. We then tested the ability of the wild type and different variants of Sg4E-BP to associate *in vitro* with GST-mIF4E (Fig 6). Using m^7^GTP-affinity chromatography (cap column), we found that wild type GST-Sg4E-BP co-purified with GST-mIF4E (Fig 6A and 6B, lanes 2), whereas the YALA variant was not retained efficiently (Fig 6A and 6B, lanes 3). It was expected that the YALA variant would not bind to cap column since this mutation in human 4E-BP precludes binding to eIF4E. Strikingly the 4xE variant that mimics the hyperphosphorylated form of Sg4E-BP co-purified as well as the 4xA variant with GST-mIF4E on cap-column (Fig 6A and 6B, lanes 5 and 4, respectively). These two variants were not retained if GST-mIF4E was omitted (Fig 6A lanes 9 and 10). This result suggests that the phosphorylation of all four residues of 4E-BP is not sufficient to prevent eIF4E binding [26]. We then decided to test the ability of the 4xE variant to competitively inhibit the association between eIF4E and eIF4G (Fig 7). Different ratios of wild type or 4xE GST-Sg-4E-BP and GST-SgIF4G were mixed before affinity purification by m^7^GTP-affinity chromatography. Both a 10-fold excess of wild type (Fig 7A) and 4xE variant (Fig 7B) efficiently lowered GST-SgIF4G binding to GST-mIF4E. Taken together, these data suggest that Sg4E-BP phosphorylated on all four residues, T36/T45/S64/T69, is functional to inhibit eIF4G/eIF4E complex formation.

**Figure 8:**
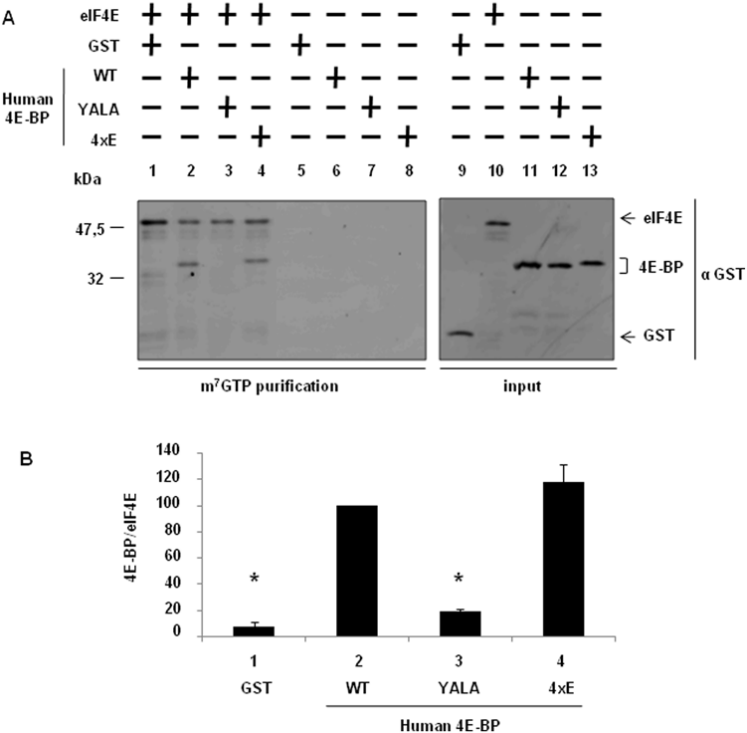
The variant mimicking hyperphosphorylation of human 4E-BP does not decrease its association with eIF4E. (A) The S/T-E human 4E-BP mutated at all four phosphorylation sites (4xE) binds to eIF4E. GST-mIF4E (eIF4E) was incubated with m^7^GTP sepharose beads (lanes 1–4) and the GST recombinant proteins were added: GST, GST-Hu4E-BP WT (WT), GST-Hu4E-BP YALA (YALA), GST-Hu4E-BP 4xE (4xE). Complexes were affinity purified and analysed by Western blot using a GST antibody. Lanes 5–8, GST-mIF4E was omitted as control for binding specificity. Inputs are shown on the right panel (lanes 9–13), they represent 10% of the volume used in the experiment. (B) Binding between the GST recombinant proteins and GST-mIF4E was analysed by quantification of the signals obtained from duplicates of two independent experiments on the Fig 8.A using Image Quant software. Error bars represent the standard deviation (s.d.) of two experiments. Significance was assessed using Fisher's *F*-test and Student's *t*-test. ^*^ *P*<0.01 significant difference between GST or GST-Hu4E-BP YALA with GST-Hu4E-BP WT.

Since the sea urchin 4xE variant could have an unanticipated function, we tested the capacity of a human 4E-BP1 variant protein mimicking hyperphosphorylation on the corresponding phosphorylation sites, T37/T46/S65/70, to bind eIF4E (Fig. 8). While the YALA variant did not associate with GST-mIF4E (lane 3), the 4xE variant (lane 4) associated in a similar manner to that of the wild type (lane 2) human 4E-BP1.

## Discussion

Using a cell-free sea urchin translation system from unfertilized eggs and early cleavage embryos, it was suggested that the activity of eukaryotic Initiation Factor 4 was repressed in unfertilized eggs owing to the presence of an unidentified inhibitor that interacted with eIF4E [38], [41]. Our previous data showed that Sg4E-BP associated with eIF4E in unfertilized eggs and that increased protein synthesis rates correlated with eIF4E/4E-BP complex dissociation in combination with phosphorylation [31] and degradation of 4E-BP [8]. Establishment of an active cell-free cap-dependent translation system allowed study of the molecular mechanisms governing the regulation of protein synthesis initiation in fertilized eggs of sea urchins. We provide here the first direct evidence that sea urchin 4E-BP efficiently inhibits protein synthesis in a cell-free cap-dependent translation system from post-fertilized eggs. These data are consistent with our hypothesis that 4E-BP is a major repressor of protein synthesis in unfertilized eggs and that its dissociation from eIF4E plays a crucial role in the increase in the rate of protein synthesis that occurs shortly following fertilization [8], [31], [42].

4E-BP sequences from protists, fungi and metazoa can be found in the Joshi/Jagus 4E-BP database (http://umbicc3-215.umbi.umd.edu). The consensus analysis from the alignment of 4E-BP from selected species suggests that 4E-BP gene duplication occurred recently in vertebrates and consequently only one 4E-BP is found per species from radial animals to echinoderms [10], [11]. To date, most functional studies of 4E-BPs have been performed in mammalian systems. In *Drosophila melanogaster*, 4E-BP exhibits a nonconsensus eIF4E-binding site and binds poorly to eIF4E [43].

*Aplysia* 4E-BP contains a consensus eIF4E-binding site, and alters cap-dependent translation when overexpressed in the soma of neurons [44]. In echinoderms, 4E-BP cDNA has not yet been cloned from starfish; however, the association of 4E-BP with eIF4E has been demonstrated to be down regulated upon meiotic maturation [45]. We show here that *Sphaerechinus granularis* 4E-BP contains a consensus eIF4E binding site and that the YALA variant does not interact with eIF4E (Fig 6). Furthermore, we provide evidence that this site is required for the cap-dependent translation inhibitory activity of the sea urchin 4E-BP (Fig 4).

We previously demonstrated that sea urchin 4E-BP is phosphorylated to a significant extent in unfertilized eggs, and that fertilization causes hyperphosphorylation [31]. These observations led us to propose that sea urchin 4E-BP phosphorylation regulates eIF4E/4E-BP complex formation in a manner similar to that of vertebrate 4E-BP1, which dissociates from eIF4E following phosphorylation of T37, T46, S65 and T70 (numbering according to human nomenclature, see introduction). Additional phosphorylation sites of mammalian 4E-BP1 have been identified on Ser 83 [46], Ser 101 [28] and Ser 112 [47]. Notably, of the seven phosphorylation sites identified in vertebrate 4E-BP1, only the four sites most linked to regulate mammalian 4E-BP binding to eIF4E are conserved in sea urchin (Fig 1) and *Aplysia* 4E-BP [44]. It was surprising to find that serine/threonine-to-glutamate mutations at all four residues on 4E-BP did not significantly decrease sea urchin 4E-BP association with eIF4E (Fig. 6A and Fig. 6B) and did not affect its ability to inhibit translation in our cell-free cap-dependent translation system (Fig. 4). The serine/threonine-to-glutamate mutations at all four residues of sea urchin 4E-BP could have a weaker effect than phosphorylation at these residues, but this hypothesis is not supported by the finding that S/T-E mutations at all four residues of *Aplysia* 4E-BP significantly abolished binding to eIF4E [44]. Therefore our data suggest that phosphorylation at the four sites of the sea urchin 4E-BP is not sufficient to release eIF4E from its repressor and are in discrepancy with the regulation of the eIF4E/4E-BP interaction via hyperphosphorylation of human 4E-BP1 and 4E-BP2 [27]. We have shown here that the human 4E-BP1 variant 4xE exhibits comparable association with eIF4E as does that of the same sea urchin 4E-BP variant. This result reinforces our suggestion that phosphorylation at T37/T46/S65/70 is not sufficient to disrupt the eIF4E/4E-BP complex and could require phosphorylation at other sites or other additional post translational modifications. It is interesting to note the presence of an acidic aspartate at position 80 in the *Aplysia* sequence (Fig. 1) which could mimic phosphorylation at the aligned S83 in human 4E-BP. A non-phosphorylatable cystein is present at this position in sea urchin 4E-BP. Therefore, we hypothesize that phosphorylation at the four sites could be required, but is not sufficient, to disrupt the 4E-BP/eIF4E complex. Functional studies have demonstrated that phosphorylation increases rigidity of the structure of 4E-BP1 [48]. Therefore, 4E-BP1 phosphorylation may regulate additional functions. In human cells, phosphorylation of 4E-BP1 not only reduces the affinity of 4E-BP1 for eIF4E, but also promotes the conversion of 4E-BP1 to poly-ubiquitinated forms that precede degradation [30]. In sea urchin, additional control of 4E-BP through rapamycin-sensitive proteolysis has been shown following egg fertilization [8], and thus phosphorylation of 4E-BP could represent an important signal for its degradation. Therefore, the finding here that the variant mimicking hyperphosphorylated 4E-BP still interacts with eIF4E and inhibits protein synthesis in a cell-free cap-dependent translation system reinforces the idea that degradation represents an important mechanism to regulate 4E-BP function in sea urchin. The proteolysis pathway responsible for 4E-BP degradation following fertilization is currently under investigation.

## Materials and Methods

### Chemicals

4-(2-aminoethyl)-benzenesulfonylfluoride hydrochloride (AEBSF) and glycine were purchased from Interchim. Acetylcholine, Sodium orthovanadate, EDTA, β-glycerophosphate, dithiothreitol (DTT), igepal, leupeptin, aprotinin, chymostatin and soybean trypsin inhibitor were obtained from Sigma. Pepstatin was purchased from Euromedex. ECL, ECL+ detection reagents, 7-methyl-GTP Sepharose 4B beads (m^7^GTP-affinity chromatography) were obtained from Amersham Pharmacia Biotech. QuickChange site-directed mutagenesis kit was obtained from Stratagene. Mouse monoclonal antibody directed against eIF4E from rabbit was purchased from Transduction Laboratories (Lexington, KY). Rabbit polyclonal antibodies directed against GST were purchased from Santa Cruz. Goat anti-mouse, and swine anti-rabbit IgG (horseradish peroxidase-coupled) were obtained from Dako SA. The mouse eIF4E recombinant construct (GST-mIF4E) was a generous gift from Simon Morley (University of Sussex, Brighton, UK). Renilla Luciferase Assay system, Riboprobe *in vitro* Transcription System and amino acids were obtained from Promega. *Eco*RV enzyme was purchased from Biolabs and RNasin was obtained from Ambion. The Smart Race cDNA amplification kit was obtained from Clontech.

### Cloning of *S. granularis Sg4E-BP*

Primers designed from the *Strongylocentrotus purpuratus* genome: F (5′- ATG TCA GCC CCT GCA GCA AGT CG -3′) and R (5′- TCA GAT GTC CAT TTG GAA CTG ATC TTC AGC -3′) were used to amplify a 342-bp fragment by PCR using a *S. granularis* cDNA library. 3′ ends of the *S. granularis* cDNA fragment were extended by RACE PCR (Smart RACE cDNA amplification kit) using primer F to obtain a final 882 nt cDNA, containing a 342 nt ORF. Sequencing was performed on an Applied Biosystems AB3130 automatic sequencer at the Génopole Ouest sequencing facility in Roscoff (France). The EMBL accession number for *S. granularis Sg4E-BP cDNA* is FM179949.

### GST-recombinant proteins

The 342-bp fragment corresponding to the *Sg4E-BP* coding region was amplified using primers 5′- CGC GGA TCC ATG TCA GCC CCT GCA GCA AGT CG -3′ and 5′- CCG CTC GAG CGG TCA GAT GTC CAT TTG GAA CTG ATC -3′. The fragment was inserted into pGEX-4T-1 vector digested with *Bam*HI and *Xho*I. The GST-Hu4E-BP wild type construct was provided by the Sonenberg lab [22]. GST-Sg4E-BP YALA (Y53A/L58A), GST-Sg4E-BP 4xA (T36A/T45A/S64A/T69A), GST-Sg4E-BP 4xE (T36E/T45E/S64E/T69E), GST-Hu4E-BP YALA (Y54A/L59A), and GST-Hu4E-BP 4xE (T37E/T46E/S65E/T70E) were produced using QuickChange site-directed mutagenesis and confirmed by DNA sequencing. Wild type and variant proteins: GST, GST-Sg4E-BP, GST-Sg4E-BP YALA, GST-Sg4E-BP 4xA, GST-Sg4E-BP 4xE, GST-SgIF4G [33], GST-Hu4E-BP, GST-Hu4E-BP YALA, GST-Hu4E-BP 4xE and GST-mIF4E were overexpressed in *E. coli* (strain BL21) and purified on a glutathione Sepharose 4B column according to the manufacturer's instructions.

### Preparation of gametes

*Sphaerechinus granularis* sea urchins were collected in the Brest area (France), kept in seawater and used within 5 days. Spawning of gametes was induced by intracoelomic injection of 0.1 M acetylcholine. Eggs were collected in 0.22 µm Millipore-filtered seawater and rinsed twice by centrifugation at 2,000 rpm for 2 min. For the preparation of lysates, eggs were dejellied by swirling twenty seconds in 3.5 mM citric acid pH 5 and rinsed three times in filtered seawater prior to fertilization. For fertilization, eggs were suspended in filtered seawater (5% suspension) containing 0.1% glycine. Diluted sperm was added to the eggs and withdrawn after fertilization envelope elevation. Experiments were only performed on batches exhibiting greater than 90% fertilization, and each experiment used gametes from a single female. Cultures of embryos were performed at 16°C under constant agitation.

### Interaction between GST-Sg4E-BP and endogenous SgIF4E

This interaction was analysed by GST pull down assay. Unfertilized or 60 min post-fertilized embryos were collected after centrifugation for 2 min. at 2,000 g. Cells were lysed by passage through a 25G syringe in one cell volume of 2× binding buffer (40 mM HEPES pH 7.4, 0.2 mM sodium orthovanadate, 100 mM NaCl, 0.4 mM EDTA, 2 mM DTT, 20 mM PPi, 100 mM sodium fluoride, 100 mM β-glycerophosphate, 1 mM AEBSF and 20 µg/ml of aprotinin and leupeptin). Cell lysates were centrifuged for 15 min at 16,000 g at 4°C in an Eppendorf centrifuge 5415R and the supernatants were stored at −20°C before use. Protein quantification was performed in duplicate by the Bradford assay. One microgram of GST-Sg4E-BP or GST alone were pre-incubated with 25 µl of glutathione-Sepharose in a final volume of 200 µl of 1× binding buffer for 1 hour at 4°C. The beads were washed three times with 1× binding buffer. Five hundred microliters (2 mg of proteins) of supernatant resulting from the 16,000 g centrifugation were mixed with the beads and incubated end-over-end for 60 min at 4°C. Then the samples were washed three times with 1 ml of 1× binding buffer containing 100 mM NaCl. Laemmli sample buffer was added directly to the beads, and the proteins were resolved by SDS-PAGE and analysed by Western blotting.

### Analysis of *in vitro* interaction between GST-4E-BP recombinant proteins and GST-mIF4E

After production and purification, GST recombinant proteins were dialysed overnight in buffer A (50 mM HEPES pH 7.7, 150 mM KCl, 1 mM EDTA, 5% glycerol). One microgram of GST-mIF4E was incubated for 1 hour with 25 µl of m^7^GTP-Sepharose beads in buffer A. After washing, the beads were incubated for 1 hour in buffer A containing 1 mg/ml of BSA, 0.5% Igepal with one microgram of GST, GST-Sg4E-BP, GST-Sg4E-BP YALA, GST-4E-BP 4xA, GST-Sg4E-BP 4xE, GST-Hu4E-BP, GST-Hu4E-BP YALA or GST-Hu4E-BP 4xE. After extensive washing, the beads were boiled in Laemmli buffer and analysed by Western blotting using anti-GST antibodies and a chemiluminescence detection system (ECL+).

### Analysis of *in vitro* competition between GST-Sg4E-BP recombinant proteins and GST-SgIF4G

After production and purification, GST recombinant proteins were dialysed overnight in buffer A (50 mM HEPES pH 7.7, 150 mM KCl, 1 mM EDTA, 5% glycerol). One microgram of GST-mIF4E was incubated for 1 hour with 25 µl of m^7^GTP-Sepharose beads in buffer A. After washing, the beads were incubated for 1 hour in buffer A, containing 1 mg/ml of BSA, 0.5% Igepal with one, two or ten micrograms of GST-Sg4E-BP, GST-Sg4E-BP 4xE or GST-SgIF4G. After extensive washing, the beads were boiled in Laemmli buffer and analysed by Western blotting using anti-GST antibodies and a chemiluminescence detection system (ECL+).

### Western blot analyses

Western blot analyses were performed following electrophoretic transfer of proteins from SDS-PAGE onto 0.22-µm nitrocellulose membranes [49]. GST fusion proteins were analysed using rabbit polyclonal antibodies directed against GST. SgIF4E was analysed using mouse monoclonal antibody directed against rabbit eIF4E (BD transduction laboratories). Membranes were incubated with antibodies directed against GST (1∶2000) or eIF4E (1∶2000) in 20 mM Tris-HCl (pH 7.6), 5% skimmed milk and 0.1% Tween 20 at room temperature. The antigen-antibody complex was measured by chemiluminescence using horseradish peroxidase-coupled secondary antibodies according to the manufacturer's instructions (ECL or ECL+).

### Sea urchin Cell-free translation system

*Sphaerechinus granularis* unfertilized eggs and 30 min post fertilization embryos were obtained as described above in “Preparation of gametes”. Fertilization was done at 16°C in sea water containing 50 mM 3-amino-1,2,4-triazole to prevent hardening of the fertilization envelope [50]. The procedure we used to prepare lysates is a modification of the method described by Winkler and Steinhardt [51] for *Lytechinus pictus* and by Lopo [37] for *Strongylocentrotus purpuratus*. Unfertilized eggs or 30 min embryos (2 ml) were washed twice in 6 vol. of ice-cold buffer (50 mM HEPES pH 7.2, 40 mM NaCl, 106 mM potassium gluconate, 263 mM glycerol, 300 mM glycine, 10 mM EGTA, 7.3 mM CaCl2, 0.52 mM MgCl2, 80 mM β-glycerophosphate) complemented with 1 mg/ml soybean trypsin inhibitor and 0.5 mg/ml reduced glutathione. Cells were then resuspended 1∶1 in the same buffer after addition of 300 units/ml RNasin, 0.1 mg/ml Leupeptin, 0.1 mg/ml Pepstatin, 0.1 µg/ml Chymostatin. Cells were lysed after fifteen strokes in a Dounce homogenizer and centrifuged for 5 min at 15,000 g at 4°C. The supernatant was frozen in liquid N2 and stored at −80°C.

After dilution to obtain the same protein concentration between unfertilized and fertilized extracts, the cell-free system was complemented with the master mix [37] to give final concentrations of 2 mM ATP, 0.8 mM GTP, 20 mM creatine phosphate, 10 units/ml of creatine phosphokinase, 50 µM glucose 6-phosphate, 2.8 mM MgCl2, 20 amino acids (50 µM), 0.1 mg/ml Leupeptin and 0.1 mg/ml Pepstatin. Buffer or GST-recombinant proteins dialysed against the ice cold buffer were added to these lysates. Addition of mRNA (0,4 fmol/µl of reaction) was considered as the start of the translation reaction. Cap and Uncapped mRNA were obtained after *in vitro* transcription with the plasmid pGb-Eg2-410Δ2-hxG-A65 linearised with *Eco*RV [52]. The final volume of the *in vitro* translation reaction is 15 µl. After 1 h at 16°C under agitation, reactions were stopped and luminescence was measured on TriStar LB 941 from Berthold Technologies.
